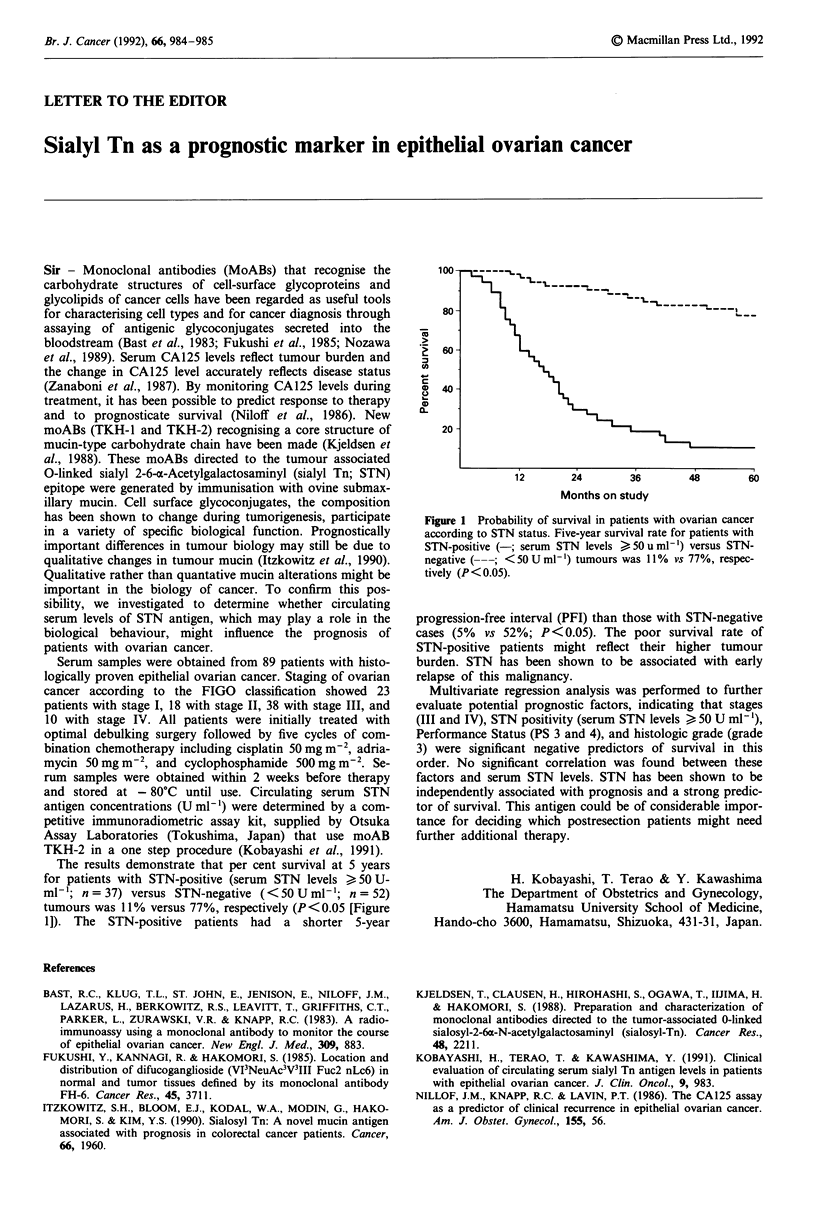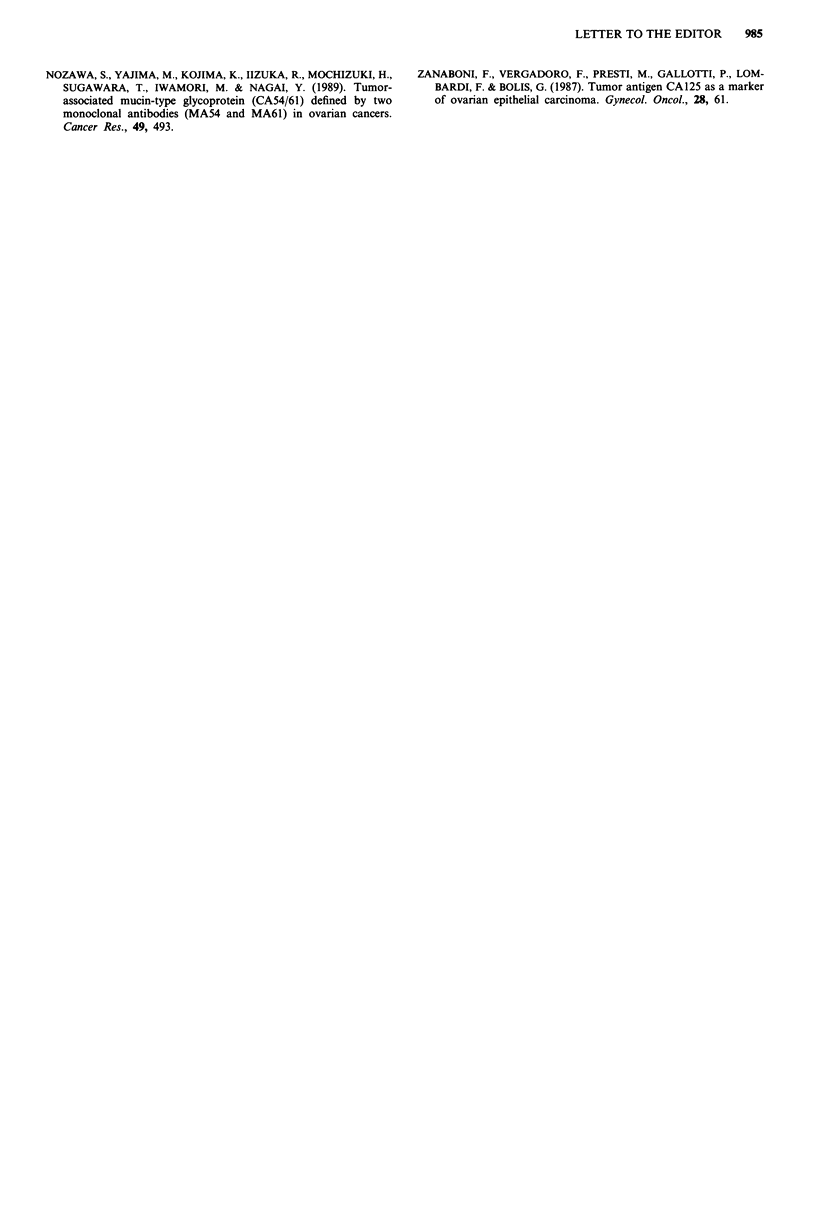# Sialyl Tn as a prognostic marker in epithelial ovarian cancer.

**DOI:** 10.1038/bjc.1992.397

**Published:** 1992-11

**Authors:** H. Kobayashi, T. Terao, Y. Kawashima


*Br. J. Cancer* (1992), **66**, 984–985 © Macmillan Press Ltd., 1992

### LETTER TO THE EDITOR

### Sialyl Tn as a prognostic marker in epithelial ovarian cancer

**Sir** – Monoclonal antibodies (MoABs) that recognise the carbohydrate structures of cell-surface glycoproteins and glycolipids of cancer cells have been regarded as useful tools for characterising cell types and for cancer diagnosis through assaying of antigenic glycoconjugates secreted into the bloodstream (Bast *et al.*, 1983; Fukushi *et al.*, 1985; Nozawa *et al.*, 1989). Serum CA125 levels reflect tumour burden and the change in CA125 level accurately reflects disease status (Zanaboni *et al.*, 1987). By monitoring CA125 levels during treatment, it has been possible to predict response to therapy and to prognosticate survival (Niloff *et al.*, 1986). New moABs (TKH-1 and TKH-2) recognising a core structure of mucin-type carbohydrate chain have been made (Kjeldsen *et al.*, 1988). These moABs directed to the tumour associated O-linked sialyl 2-6-α-Acetylgalactosaminyl (sialyl Tn; STN) epitope were generated by immunisation with ovine submaxillary mucin. Cell surface glycoconjugates, the composition has been shown to change during tumorigenesis, participate in a variety of specific biological function. Prognostically important differences in tumour biology may still be due to qualitative changes in tumour mucin (Itzkowitz *et al.*, 1990). Qualitative rather than quantative mucin alterations might be important in the biology of cancer. To confirm this possibility, we investigated to determine whether circulating serum levels of STN antigen, which may play a role in the biological behaviour, might influence the prognosis of patients with ovarian cancer.

Serum samples were obtained from 89 patients with histologically proven epithelial ovarian cancer. Staging of ovarian cancer according to the FIGO classification showed 23 patients with stage I, 18 with stage II, 38 with stage III, and 10 with stage IV. All patients were initially treated with optimal debulking surgery followed by five cycles of combination chemotherapy including cisplatin 50 mg m$^{-2}$, adriamycin 50 mg m$^{-2}$, and cyclophosphamide 500 mg m$^{-2}$. Serum samples were obtained within 2 weeks before therapy and stored at −80°C until use. Circulating serum STN antigen concentrations (U ml$^{-1}$) were determined by a competitive immunoradiometric assay kit, supplied by Otsuka Assay Laboratories (Tokushima, Japan) that use moAB TKH-2 in a one step procedure (Kobayashi *et al.*, 1991).

The results demonstrate that per cent survival at 5 years for patients with STN-positive (serum STN levels $\geqslant$50 U-ml$^{-1}$; $n = 37$) versus STN-negative ($<$50 U ml$^{-1}$; $n = 52$) tumours was 11% versus 77%, respectively ($P<0.05$ [Figure 1]). The STN-positive patients had a shorter 5-year

**Figure 1** Probability of survival in patients with ovarian cancer according to STN status. Five-year survival rate for patients with STN-positive (—; serum STN levels $\geqslant$50 u ml$^{-1}$) versus STN-negative (---; $<$50 U ml$^{-1}$) tumours was 11% *vs* 77%, respectively ($P<0.05$).

progression-free interval (PFI) than those with STN-negative cases (5% *vs* 52%; $P<0.05$). The poor survival rate of STN-positive patients might reflect their higher tumour burden. STN has been shown to be associated with early relapse of this malignancy.

Multivariate regression analysis was performed to further evaluate potential prognostic factors, indicating that stages (III and IV), STN positivity (serum STN levels $\geqslant$50 U ml$^{-1}$), Performance Status (PS 3 and 4), and histologic grade (grade 3) were significant negative predictors of survival in this order. No significant correlation was found between these factors and serum STN levels. STN has been shown to be independently associated with prognosis and a strong predictor of survival. This antigen could be of considerable importance for deciding which postresection patients might need further additional therapy.

H. Kobayashi, T. Terao & Y. Kawashima
The Department of Obstetrics and Gynecology,
Hamamatsu University School of Medicine,
Hando-cho 3600, Hamamatsu, Shizuoka, 431-31, Japan.

### References

BAST, R.C., KLUG, T.L., ST. JOHN, E., JENISON, E., NILOFF, J.M., LAZARUS, H., BERKOWITZ, R.S., LEAVITT, T., GRIFFITHS, C.T., PARKER, L., ZURAWSKI, V.R. & KNAPP, R.C. (1983). A radioimmunoassy using a monoclonal antibody to monitor the course of epithelial ovarian cancer. *New Engl. J. Med.*, **309**, 883.

FUKUSHI, Y., KANNAGI, R. & HAKOMORI, S. (1985). Location and distribution of difucoganglioside (VI$^3$NeuAc$^3$V$^3$III Fuc2 nLc6) in normal and tumor tissues defined by its monoclonal antibody FH-6. *Cancer Res.*, **45**, 3711.

ITZKOWITZ, S.H., BLOOM, E.J., KODAL, W.A., MODIN, G., HAKOMORI, S. & KIM, Y.S. (1990). Sialosyl Tn: A novel mucin antigen associated with prognosis in colorectal cancer patients. *Cancer*, **66**, 1960.

KJELDSEN, T., CLAUSEN, H., HIROHASHI, S., OGAWA, T., IIJIMA, H. & HAKOMORI, S. (1988). Preparation and characterization of monoclonal antibodies directed to the tumor-associated 0-linked sialosyl-2-6α-N-acetylgalactosaminyl (sialosyl-Tn). *Cancer Res.*, **48**, 2211.

KOBAYASHI, H., TERAO, T. & KAWASHIMA, Y. (1991). Clinical evaluation of circulating serum sialyl Tn antigen levels in patients with epithelial ovarian cancer. *J. Clin. Oncol.*, **9**, 983.

NILLOF, J.M., KNAPP, R.C. & LAVIN, P.T. (1986). The CA125 assay as a predictor of clinical recurrence in epithelial ovarian cancer. *Am. J. Obstet. Gynecol.*, **155**, 56.

NOZAWA, S., YAJIMA, M., KOJIMA, K., IIZUKA, R., MOCHIZUKI, H., SUGAWARA, T., IWAMORI, M. & NAGAI, Y. (1989). Tumor-associated mucin-type glycoprotein (CA54/61) defined by two monoclonal antibodies (MA54 and MA61) in ovarian cancers. *Cancer Res.*, **49**, 493.

ZANABONI, F., VERGADORO, F., PRESTI, M., GALLOTTI, P., LOMBARDI, F. & BOLIS, G. (1987). Tumor antigen CA125 as a marker of ovarian epithelial carcinoma. *Gynecol. Oncol.*, **28**, 61.